# Coccidioidomycosis in Allogeneic Stem Cell Transplant Recipients: Case Series and Review of the Literature

**DOI:** 10.3390/jof7050339

**Published:** 2021-04-27

**Authors:** Christopher F. Saling, Juan Gea-Banacloche, John S. Trickett, Janis E. Blair

**Affiliations:** 1 Division of Infectious Diseases, Mayo Clinic, Phoenix, AZ 85054, USA; saling.christopher@mayo.edu (C.F.S.); gea-banacloche.juan@mayo.edu (J.G.-B.); 2 Department of Internal Medicine, Mayo Clinic, Phoenix, AZ 85054, USA; trickett.john@mayo.edu

**Keywords:** coccidioidomycosis, *Coccidioides*, allogeneic stem cell transplant, bone marrow transplant

## Abstract

*Coccidioides* is an endemic fungus of the Southwest United States that causes the disease coccidioidomycosis. Immunocompromised persons are at increased risk for severe infection and dissemination. One such population is allogeneic bone marrow transplant (allo-HCT) recipients, but accounts of coccidioidal infection in these patients have rarely been documented. We present two cases of *Coccidioides* in allo-HCT recipients with good outcomes: one patient who developed pulmonary coccidioidomycosis in the late post-engraftment phase and another with known controlled disseminated infection at the time of transplant. A review of the literature identified 19 allo-HCT recipients with coccidioidomycosis. Due to the limited published literature, no guidelines have yet been established regarding optimal prophylaxis and treatment of *Coccidioides* infection in allo-HCT recipients. Candidates for transplantation should undergo a rigorous pre-transplant assessment to identify evidence of prior or active coccidioidomycosis. In our experience, patients who visit or live in *Coccidioides*-endemic areas should receive primary prophylaxis for at least the first 100 days post-transplant, and duration should be extended as long as the patient remains on immunosuppression. Those with prior infection should receive secondary prophylaxis while immunosuppressed. Patients with active infection should have treatment and stabilization of infection and continue anti-fungal treatment through immunosuppression.

## 1. Introduction

*Coccidioides immitis* and *Coccidioides posadasii*, referred to collectively as *Coccidioides*, inhabit the soil in areas with arid climates and is endemic to particular areas of the Southwestern and Western United States and Central and South America [1,2]. *Coccidioides* is responsible for the disease coccidioidomycosis, also known colloquially as Valley Fever. Infection occurs after inhaling arthroconidia made airborne by soil disturbance [1]. The incubation period for coccidioidomycosis ranges from 1 to 3 weeks [3]. Approximately 60% of primary infections are asymptomatic, with the other 40% demonstrating a range of symptoms, from mild flu-like to severe pulmonary or extrapulmonary infection [4]. Pulmonary coccidioidomycosis is a common manifestation in people with primary infection and often resolves with a residual pulmonary nodule or cavity; rarely, this infection manifests as a progressive pneumonia [4,5]. Extrapulmonary dissemination can occur as a result of lymphatic spread outside of the lungs, and the burden of disease can be devastating [4,6]. Common areas for dissemination are the central nervous system, bones, and joints, but virtually any tissue can be affected [4,6]. Less than 1% of primary infection will disseminate [7].

In primary coccidioidomycosis, most arthroconidia escape the initial innate immune system response and move into the terminal bronchioles, where they transform in size and shape to become spherules [4,7,8]. Phagocytosis of spherules is especially difficult due to their large size and outer extracellular fibrillar matrix [8]. Each spherule in turn releases hundreds of endospores, which further increases the burden on innate immune cells [8]. Thus, it is the adaptive immune system, more specifically the T-cell response, that is paramount in controlling the disease [9]. Activated T cells release cytokines that bolster innate cell trafficking and phagocytic function, and more importantly, aid in the formation of granulomas that contain spherules and endospores [4,7,8,9]. Immunocompromised patients, especially those with impairment of cellular immunity, are at an increased risk for extrapulmonary dissemination [6,10,11].

Allogeneic hemopoietic stem cell transplant (allo-HCT) recipients are at risk for severe or extrapulmonary coccidioidomycosis [12]. However, literature describing coccidioidal disease in this population is particularly scant. We present two cases of coccidioidomycosis in allo-HCT recipients, one of whom developed acute pulmonary coccidioidomycosis after all immunosuppression was stopped, and another with controlled disseminated infection at the time of transplant. Both patients had good outcomes. We also provide a review of the medical literature regarding presentations, treatments, and outcomes of coccidioidomycosis in allo-HCT recipients.

## 2. Case Reports and Results

### 2.1. Case 1

A 61-year-old female with past medical history significant for acute myelogenous leukemia (AML) received her AML care at our institution in Phoenix, Arizona. She underwent induction chemotherapy with the “7 + 3” regimen, consisting of 7 days of cytarabine and 3 days of doxorubicin. This was followed by two cycles of consolidation therapy with busulfan and cyclophosphamide. One year after her AML diagnosis, the patient underwent allogeneic mismatched, unrelated stem cell transplantation with busulfan and cyclophosphamide conditioning. Pre-transplant serum *Coccidioides* antibodies via enzyme immunoassay (EIA) were negative. She received tacrolimus 0.5 milligrams (mg) daily. Her anti-fungal prophylaxis strategy included fluconazole 400 mg daily. Ultimately, she achieved complete chimerism, and all immunosuppression was stopped approximately 2 years after transplant. Although the patient lived in Montana, she routinely traveled to our institution in Arizona for follow-up.

**Figure 1:**
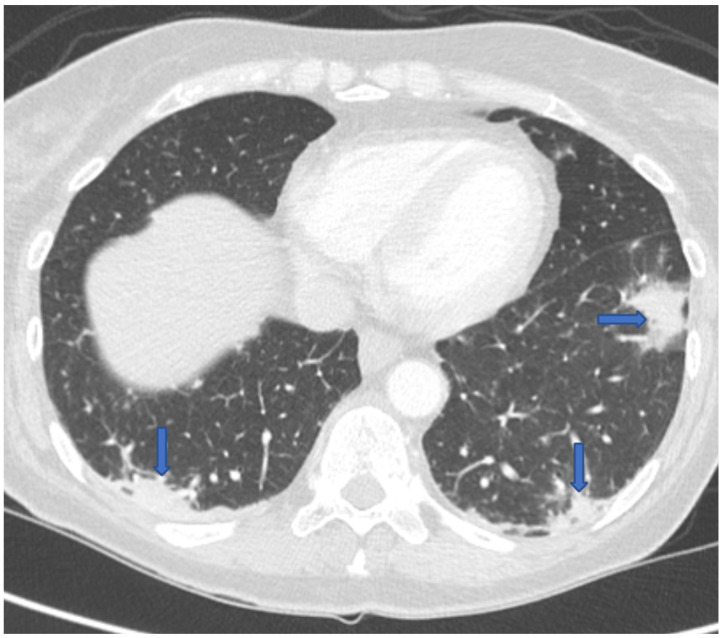
CT chest revealing multifocal bilateral peripheral opacities.

Eighteen months following her transplant, she went to her local hospital in Montana with 3 months of progressive fatigue, 2 weeks of dyspnea and dry cough, and recent onset of drenching night sweats and headaches. She had been off immunosuppression for 6 months and anti-coccidioidal prophylaxis for 3 months. A chest computed tomography (CT) scan revealed bilateral ground-glass and nodular lung opacities in a peripheral distribution (Figure 1). She was diagnosed with a community acquired pneumonia and discharged home on oral azithromycin. A few days after homegoing, the patient developed an erythematous maculopapular rash over her face and arms, thought to be an allergic reaction. Since the patient had no improvement in her symptoms, she decided to travel to our institution for further care.

In the emergency room, the patient was afebrile and normotensive. She had no respiratory distress, and her oxygen saturation was 99% on room air. Examination identified diffuse crackles on inspiration and no visible skin lesions, which had resolved spontaneously. White blood cell count was 10.7 × 10^9^/L with an absolute neutrophil count of 8.72 × 10^9^/L and no peripheral eosinophilia. An extensive microbiological workup for pneumonia, including opportunistic pathogens, was performed. Urine *Legionella* and *Streptococcus pneumonia* antigens were done and were negative. Sputum bacterial and fungal cultures also returned negative. Serum *Coccidioides* immunoglobulin M (IgM) EIA was negative and immunoglobulin G (IgG) EIA was positive. Serum *Coccidioides* IgM and IgG by immunodiffusion (ID) were equivocal and negative, respectively. *Coccidioides* IgG by complement fixation (CF) was negative. Bronchoscopy with bronchoalveolar lavage (BAL) was subsequently performed, yielding negative *Pneumocystis* smear and polymerase chain reaction (PCR), *Legionella* culture, *Nocardia* smear, *Aspergillus* antigen, mycobacterial smear, fungal cultures, and *Coccidioides* PCR.

Based on typical symptoms, radiographic abnormalities, and seroconversion of the coccidioidomycosis IgG by EIA, a diagnosis of probable acute pulmonary coccidioidomycoses was made. We believe that she contracted her coccidioidomycosis in the time she stopped anti-fungal prophylaxis while visiting our institution, located in a *Coccidioides*-endemic region. Examination did not identify any extrapulmonary infection. She was treated with oral fluconazole 400 mg daily with close outpatient follow-up. Repeat *Coccidioides* serologies were performed 1 month later; IgG EIA remained positive, and IgM by ID became positive. Nine months after diagnosis and treatment, chest imaging showed complete resolution of opacities and serology was positive only for IgG by EIA. After 10 months of treatment, fluconazole was stopped. The patient has remained off therapy now for 20 months with no relapse of her coccidioidomycosis.

### 2.2. Case 2

A 58-year-old male from Arizona with past medical history significant for chronic lymphocytic leukemia (CLL), chronic active hepatitis B, diabetes mellitus, idiopathic thrombocytopenic purpura (ITP) requiring intravenous immunoglobulin (IVIG) and intermittent corticosteroids, and remote pulmonary coccidioidomycosis. His CLL was diagnosed 15 months prior, and he had undergone four cycles of chemotherapy (fludarabine, cyclophosphamide, and rituximab); the last cycle was completed a few days prior to his presentation to our institution with 3 days of fever, rigors, dry cough, and dyspnea. He denied any headache or rash. Upon presentation, his temperature was 38.7 degrees Celsius and oxygen saturation 94% on room air. White blood cell count was 2.6 × 10^9^/L with no eosinophils on differential. Hemoglobin and platelet count were 5.7 g/dL and 23 × 10^9^/L, respectively. Chest CT showed interval development of diffuse tree-in-bud opacities more severe in the upper lobes. He was treated empirically with levofloxacin and fluconazole. Bronchoscopy with BAL was positive for rhinovirus PCR. Serum *Coccidioides* serology by EIA, ID, and CF were all negative. His fevers resolved, and he was discharged to complete a 7-day course of levofloxacin.

One week later, he returned to our institution with complaints of headache and blurred vision for 3 days, and his fever recurred. Magnetic resonance imaging (MRI) brain scan revealed leptomeningeal enhancement and multiple small hemorrhagic lesions within the bilateral cerebral and cerebellar hemispheres, right medulla, and left pons. Chest CT showed significant improvement in pulmonary infiltrates. Serum beta-D glucan was positive at 126 pg/mL. Lumbar puncture (LP) showed 40 erythrocytes and 42 total nucleated cells with 55% neutrophils, 42% lymphocytes, 3% monocytes, total protein 122 mg/dL, and glucose 21 mg/dL. CSF coccidioidal serology and fungal culture were negative, but the *Coccidioides* PCR was positive. Blood cultures grew *Coccidioides*. He was treated with liposomal amphotericin 5 mg/kg daily plus fluconazole 800 mg daily, and clinically improved. Liposomal amphotericin was administered for 2 weeks, and the patient was discharged on fluconazole 800 mg daily.

After discharge, the patient remained pancytopenic and transfusion dependent. Bone marrow biopsy revealed hypocellular marrow without evidence for dysplasia. Infectious disease was consulted to evaluate his candidacy for allo-HCT. Eight months following the diagnosis of *Coccidioides* meningitis, serum beta-D glucan and *Coccidioides* serum serologies by EIA, ID, and CF were negative, repeat chest CT was normal, and MRI brain scan showed resolution of leptomeningeal enhancement. Bone scan was also normal. The patient subsequently underwent allogeneic mismatched, unrelated stem cell transplantation with fludarabine, carmustine, and melphalan conditioning. Standard doses of methotrexate and tacrolimus were given for graft versus host disease (GVHD) prophylaxis for 1 month. He continued fluconazole 800 mg daily. His post-transplant course was complicated by GVHD at 11 months, which required high dose prednisone and tacrolimus. Serial *Coccidioides* serologies remain negative, the patient continues fluconazole, and has no symptoms of reactivated coccidioidomycosis for 3.5 years since allo-HCT.

## 3. Discussion

Allo-HCT is a medical procedure by which the myelopoietic and immune system of a recipient are replaced by infusing hematopoietic stem cells from a donor [13,14]. The stem cells may be obtained by direct harvest of the donor’s bone marrow (in which case the proper name is “bone marrow transplant” (BMT)) or by mobilizing the stem cells in the donor by administering granulocyte-colony-stimulating factor or plerixafor and then collecting them from the peripheral blood by lymphocyte apheresis [13,14]. In this latter case, the appropriate term is peripheral blood stem cells (PBSC) transplant [13,14].

Indications for allo-HCT include some hematologic malignancies (most prominently AML but also chronic myelogenous leukemia (CML), acute lymphocytic leukemia (ALL) and selected cases of lymphoma and CLL) and some non-malignant conditions like immunodeficiency, aplastic anemia, and hemoglobinopathies [15]. The procedure itself involves elimination of the recipient’s hematopoietic and lymphoid system by a “conditioning regimen” that includes chemotherapy, radiation, and/or immunoablative therapies (e.g., anti-thymocyte globulin), followed by infusion of the donor’s stem cells and prolonged immunosuppression to prevent the donor’s immune system from reacting against the recipient (also known as GVHD) [16,17,18]. In most cases, the immunosuppression administered to prevent GVHD may be tapered after about 100 days and eventually completely discontinued about 6 months after the infusion of stem cells [18]. Ideally, the recipient has then achieved “stable chimerism”, a state where their myeloid and lymphoid cells are donor-derived but there is no rejection, nor GVHD [18].

A variety of allo-HCT types may be distinguished based on different criteria: the degree of identity between donor and recipient according to Human Leucocyte Antigen (HLA)-typing (e.g., matched sibling donor, matched unrelated donor, haploidentical donor), conditioning regimen (myeloablative vs. non-myeloablative), source of stem cells (bone marrow, PBSC, cord blood), and type of GVHD prophylaxis (in vitro or in vivo T-cell depletion, cyclophosphamide-based, tacrolimus-methotrexate based, and others) [17,18,19]. Different kinds of allo-HCT have distinct risks and indications that are beyond the scope of this summary. The important concept from the standpoint of infectious diseases is that, when the transplant has no complications, the immunosuppression is discontinued at about six months, and then the new immune system reconstitutes from the donor’s stem cells, which will differentiate into T and B lymphocytes and mature and develop in the bone marrow and thymus [18,20]. There may be profound individual differences between recipients in terms of completeness of immune reconstitution (e.g., depending on age the thymus may not be able to provide adequate maturation of the new T cells), but as a rule, allo-HCT recipients start receiving live vaccines two years after the procedure, provided they have no active GVHD and are not receiving immunosuppressive medication [21].

The risk of infection after allo-HCT varies with time. Traditionally, several distinct phases have been contemplated: “pre-engraftment”(less than 30 days after transplant), when the primary risk factors are the pre-existing condition that prompted the transplant and the mucositis and neutropenia induced by chemotherapy; “early post-engraftment”(between 30 days and 100 days after transplant), when cellular immunity is depressed by the immunosuppressive agents administered to prevent or treat graft versus host disease; and “late post-engraftment” (more than 100 days after transplant), when the immunosuppression is tapered and immune reconstitution is proceeding [22]. This framework helps to predict bacterial, viral, and fungal infections. If GVHD develops and requires further immunosuppression, further opportunistic infections may ensue. The standard use of anti-fungal treatment and prophylaxis has demonstrated lower rates of coccidioidal infection in allo-HCT patients [23].

The types of hematological transplant influence the infectious risk. The conditioning regimen may be more myeloablative (resulting in longer neutropenia and more severe mucositis) or more “immunoablative”, potentially increasing the risk of latent viral infections later on [20]. Transplants using PBSCs contain 17 times more mature cells than transplants using bone marrow. The increased numbers of mature cells may potentially result in more GVHD, but also in more memory T cells. Some data suggest an increased rate of infections after BMT [24]. In the case of cord blood transplants, the lack of any transmission of memory cells results in a greatly increased risk of infections controlled by T cells, particularly viral infections [25].

Against this background, coccidioidomycosis is a particularly interesting infection. At the time of allo-HCT, a patient may have either an absence or true presence (known or unknown) of pre-existing *Coccidioides* infection. Since the immune mechanisms to gain control of primary infection (i.e., control of the growth and proliferation of early infection) are different from the ones required to keep it controlled (granuloma maintenance) [4,7,8,9], one could anticipate different risk periods and potentially differing preventive strategies. For instance, it is unclear how important neutrophils are to control *Coccidioides*. As opposed to aspergillosis, whose risk increases rapidly after three weeks of neutropenia [26], coccidioidomycosis appears to be unaffected by this circumstance [7]. On the other hand, whereas the risk of aspergillosis is insignificant during the late phase unless the patient is receiving corticosteroids or other immunosuppressive agents [26], *Coccidioides* is an ever-present risk in endemic areas and effective immune responses may reconstitute slowly following allo-HCT.

The medical literature addressing coccidioidomycosis in allo-HCT recipients is limited. In addition to our 2 cases, we found 19 reports of coccidioidomycosis in allo-HCT recipients [23,27,28,29,30,31]. Table 1 summarizes eight cases (including our two cases) [27,28,29,30]. The table does not include two cases of pulmonary coccidioidomycosis in allo-HCT identified through the Transplant-Associated Surveillance Network between March 2001 and March 2006 since no specific information regarding these patients was provided [3]). In addition, a retrospective review of allo-HCT recipients between January 2003 and September 2013 from a single institution in the Phoenix, AZ metropolitan area, reported an additional 11 patients (incidence rate of 2.6%) but did not provide adequate individual details to include in the table [23]. One of these patients had active coccidioidomycosis at the time of allo-HCT and died. Three of the eleven patients had reactivation of coccidioidal infection. One patient developed *Coccidioides* in the early post-engraftment phase and nine patients developed coccidioidomycosis in the late post-engraftment phase. The time of active coccidioidomycosis from allo-HCT ranged between Day 0 and 48 months. Seven of the eleven patients were receiving immunosuppressive therapy at the time of coccidioidomycosis diagnosis, and two of these seven patients received concurrent anti-fungal prophylaxis. Five of the eleven (45%) patients died from active coccidioidomycosis. No patients who developed coccidioidomycosis more than 2 years post-allo-HCT died [23].

Of the eight patients in Table 1 and Table 2, Patients 4, 6, and 8 had active coccidioidomycosis at time of transplant [28,30]. One of these three patients had disseminated infection and died (Patient 6) [30]. Patient 4 had known pulmonary coccidioidomycosis with improving pulmonary infiltrates on fluconazole at time of transplant and survived [28]. Patient 8 is our patient with known *Coccidioides* meningitis at time of allo-HCT whose clinical course is outlined in Case 2. Two of the total eight patients developed *Coccidioides* infection in the pre-engraftment phase (Patients 1 and 2) [27]. Neither of these two patients were receiving any anti-fungal prophylaxis at the time of allo-HCT, and one died (Patient 1) despite treatment with amphotericin B. One patient (Patient 5) had immunosuppression increased due to GVHD and developed extra-thoracic *Coccidioides* infection in the early post-engraftment phase 30 days after fluconazole prophylaxis was discontinued [29]. This patient died. Two of the eight patients developed coccidioidomycosis in the late post-engraftment phase (Patients 3 and 7) [27]. Patient 3 had a post-transplant course complicated by GVHD and developed disseminated coccidioidomycosis at Day +296 and died. This patient was not receiving any anti-fungal prophylaxis. Patient 7 is described earlier in the present paper.

Due to the limited number of cases, much ambiguity exists regarding the optimal prophylaxis and treatment of coccidioidomycosis in allo-HCT recipients. What follows is our expert opinion regarding *Coccidioides* screening, primary and secondary *Coccidioides* prophylaxis, and treatment of active coccidioidomycosis in this patient population.

Pretransplant evaluation. It is essential that allo-HCT candidates undergo a thorough pre-transplant assessment to evaluate prior exposure to *Coccidioides* and to identify and treat active coccidioidomycosis. This assessment starts with a detailed travel history. Patients who have traveled to or have resided in a *Coccidioides*-endemic region should undergo serologic *Coccidioides* testing by EIA, ID, and CF [32,33,34]. If serologies are negative and the patient continues to reside in or travel to a *Coccidioides*-endemic region, it may be prudent to initiate coccidioidomycosis-specific prophylaxis. It is the practice of our institution, located in a high-*Coccidioides*-endemic area, to give fluconazole 400 mg daily for the first 100 days following allo-HCT. This should be extended beyond 100 days if the patient requires continued immunosuppression.

Pre-transplant evaluation and prophylaxis for patients with prior coccidioidomycosis. For patients with positive *Coccidioides* serologies, a baseline chest CT could be considered for future comparison. Ideally, those with active disease should not undergo allo-HCT until the infection is well controlled (i.e., resolution of symptoms and pulmonary infiltrates and low or negative CF titers) since mortality from active infection at the time of allo-HCT is 50% [23,28,30]. There is no consensus regarding dose or duration of antifungal prophylaxis in those with prior coccidioidomycosis, but we recommend these medications not be stopped until the patient is off all immunosuppression due to risk for reactivation. Since GVHD requires increased and prolonged immunosuppression, such patients would likely benefit from prolonged anti-fungal prophylaxis. For this purpose, we recommend fluconazole 400 mg daily; itraconazole, voriconazole, posaconazole, or isavuconazole could be used as alternatives. During follow-up visits, assessment for symptoms and serial *Coccidioides* serologies should be monitored. If there are symptoms suspicious for coccidioidomycosis, newly positive *Coccidioides* serologies, or a rising CF titer, a chest CT and bronchoscopy with BAL sent for fungal culture and *Coccidioides* PCR should be considered. Urine *Coccidioides* antigen testing could also be considered [32]. *Coccidioides* serologies should be performed at initial evaluation and repeated in 4-6 weeks to improve detection [32].

Active coccidioidomycosis at time of allo-HCT. Based on the current review as well as our Case 2, patients with active coccidioidomycosis at the time of allo-HCT experience 50% mortality [23,28,30]. Poor outcomes were associated with uncontrolled coccidioidomycosis or unbeknownst active *Coccidioides* infection at the time of transplant. Patients who survived received anti-*Coccidioides* treatments with radiographic and symptomatic improvement prior to transplant. When possible, it is our recommendation that allo-HCT should not be considered in those with active coccidioidomycosis until receipt of adequate anti-fungal therapy with improvement and stabilization of their *Coccidioides* infection (improvement in symptoms, shrinking pulmonary infiltrates, and negative or decreasing CF titers). Such stabilization may require multiple months of anti-fungal treatment. Furthermore, as evidenced by our Case 2, disseminated coccidioidomycosis is not an absolute contraindication for allo-HCT, which can be performed successfully as long as the infection is well controlled and anti-fungal treatment is continued. Because the mortality is high, each candidate for transplantation must be meticulously reviewed, weighing the risk and benefit, on a case-by-case basis.

Evaluation and treatment of active coccidioidomycosis at any phase of allo-HCT. For patients who develop active coccidioidomycosis after allo-HCT, either by primary infection or reactivation, a thorough history and physical exam should be performed to assess for extrapulmonary dissemination. Prolonged headache or focal deficits warrant a lumbar puncture and MRI brain scan to exclude coccidioidal meningitis. New or progressive musculoskeletal or joint pain may indicate osteomyelitis or septic arthritis, prompting consideration of a screening bone scan. Furthermore, in some laboratories a CF titer > 1:32 may warrant further exploration for possible dissemination [35].

Depending on severity or rapidity of infection progression, pulmonary or extrapulmonary coccidioidomycosis may require initial treatment with intravenous lipid-associated amphotericin until the patient stabilizes clinically and can be safely bridged to an azole [36]. Patients with stable coccidioidomycosis can be safely treated with fluconazole [36].

Fluconazole 400–800 mg daily is first-line treatment, and higher doses are generally reserved for those with meningeal dissemination [36]. Reasons to consider other azoles include the development of adverse events, lack of clinical improvement, progressing infection, or need for treatment or prophylaxis of concurrent mold infection. If there is treatment failure, a serum fluconazole trough should be checked for therapeutic levels. Other azole options to treat active pulmonary coccidioidomycosis include itraconazole, voriconazole, posaconazole, or isavuconazole [36,37]. Notably, echinocandins have minimal activity against *Coccidioides* and should not be used as monotherapy for coccidioidomycosis. Anti-coccidioidal agents are listed in Table 2.

Duration of treatment of active coccidioidomycosis. Allo-HCT recipients who develop pulmonary coccidioidomycosis without evidence for dissemination should remain on treatment as long as they remain on immunosuppression. The largely fungistatic properties of azoles prohibit fungal growth but rely on a functional immune system to eradicate infection [38]. However, there is no consensus regarding prolonging therapy once the patient achieves stable chimerism with immune reconstitution and discontinuation of immunosuppression. Similarly, there is no unanimity for treatment duration in allo-HCT recipients who develop primary coccidioidomycosis after their course of immunosuppression has been completed. Such patients should be monitored closely with serial *Coccidioides* serologies, chest imaging, and clinical signs for worsening disease. It is our opinion that treatment of active pulmonary coccidioidomycosis (with no evidence of dissemination) in allo-HCT recipients should be extended at least 6–12 months after immunosuppression is stopped. After this time, in the absence of data, if the patient is asymptomatic with resolved pulmonary infiltrates and a negative coccidioidal CF, we believe that anti-fungal treatment can be safely stopped as long as the patient continues to be monitored closely. For patients with coccidioidal meningitis we recommend lifelong treatment [36]. For patients with non-meningeal dissemination, prolonged or lifelong treatment may be necessary, but discontinuation could be considered on a case-by-case basis, depending on extent and resolution of infection, normal immune function, and ability to comply with close follow-up.

In conclusion, despite the risk of infection in such a vulnerable population, the incidence of active coccidioidomycosis in allo-HCT recipients appears to be low. In addition to our two cases, our review of the literature identified 19 other cases. This observation is likely explained by routine anti-fungal prophylaxis given during induction and following transplantation. That being said, coccidioidomycosis can be especially devasting in allo-HCT recipients. In this discussion, we have provided literature-informed guidance, but many questions still remain: Do specific conditioning regimens increase the risk for *Coccidioides* infection? Are allo-HCT patients more prone to develop coccidioidomycosis depending on engraftment phase? Does prolonged neutropenia in allo-HCT recipients increase the risk of coccidioidomycosis or correlate with worse outcomes in those with active infection? Additional study is also needed to identify data-driven recommendations for optimal duration of *Coccidioides* prophylaxis and treatment in this patient population.

## Figures and Tables

**Table 1:** Prior described cases of coccidioidomycosis in allo-HCT recipients.

Patient Number (Reference)	Conditioning Regimen	Immunosuppression at Time of Coccidioidomycosis Onset	GVHD	Coccidioidomycosis Prior to allo-HCT	Antifungal Prophylaxis	Neutropenia	Days from Transplant to Coccidioidomycosis	Probable or Proven *	Extrathoracic Dissemination	Treatment	Outcome
**Patient #1** (Reference [27])	High dose chemotherapy conditioning (unknown agent).	Unknown	NO	NO	NO	NO	+18	Proven	NO	Total dose of 2.1 g amB	Death
**Patient #2** (Reference [27])	High dose chemotherapy conditioning (unknown agent).	Unknown	NO	NO	NO	YES	+11	Proven	NO	Fluconazole 200 mg daily for 12 days; switched to amB 0.5/mg/kg 7 days; received total of 1 g amB started 42 days later.	Initial improvement. Off treatment for 42 days with relapsed coccidioidal pneumonia. Resolved after second treatment with no recurrence.
**Patient #3** (Reference [27])	High dose chemotherapy conditioning (unknown agent).	Cyclosporine and prednisone (unknown dose)	YES	NO	NO	NO	+296	Proven	YES	Total dose of 15 mg amB	Death
**Patient #4** (Reference [28])	Busulfan and total body irradiation.	Cyclosporine and mycophenolate	YES	Active disease at allo-HCT.	Fluconazole 400 mg maintenance; Lipo amB 5 mg/kg/day for total of 1.5 g prior to transplant.	N/A	+0	Probable	NO	Lipo amB 5 mg/kg/day for 55 days. Itraconazole maintenance while on steroids.	Resolved without recurrence.
**Patient #5** (Reference [29])	Cyclophosphamide	Cyclosporine, methotrexate, prednisone 140 mg daily	YES	NO	Fluconazole 400 mg at conditioning followed by 200 mg daily for 30 days.	NO	+77	Proven	YES	Unknown	Death
**Patient #6** (Reference [30])	Unknown	Rituximab	NO	Likely active at time of allo-HCT.	Voriconazole (no dose provided)	N/A	+0	Proven	YES	8 months of voriconazole (no dose provided); stopped therapy for 2 weeks; amB for 7 days and then switched back to voriconazole	Pulmonary infiltrates improved after 8 months of voriconazole. Treatment was then stopped when immuno-suppression was discontinued. Patient relapsed 2 weeks after treatment and died.
Saling et al. (Case 1)	Cytarabine and doxorubicin. Consolidation with busulfan and cyclophosphamide.	Tacrolimus stopped 6 months prior to onset of infection.	NO	NO	Fluconazole 400 mg for first 100 days post transplant.	NO	15 Months	Probable	NO	Fluconazole 400 mg for 10 months.	Resolved without recurrence.
Saling et al. (Case 2)	Fludarabine, carmustine, and melphalan	N/A	YES	Active disease at allo-HCT.	Fluconazole 800 mg daily for meningitis	N/A	+0	Proven	YES	Fluconazole 800 mg	No reactivation

Abbreviations: Allo-HCT: allogeneic hemopoietic stem cell transplant; AmB: amphotericin B; Lipo AmB: liposomal amphotericin B; GVHD: graft versus host disease; g: grams; kg: kilograms; mg: milligrams; N/A: not applicable. * Proven coccidioidomycosis: Culture positive for *Coccidioides*, polymerase chain reaction positive for *Coccidioides*, or histopathology showing *Coccidioides* spherules. * Probable coccidioidomycosis: Patients with compatible symptoms, radiographic abnormalities and positive *Coccidioides* serology.

**Table 2:** Anti-coccidioidal agents.

Anti-Fungal Agent	Dose
Fluconazole *	400–800 mg po/IV daily
Voriconazole *	Weight ≥ 40 kg: 400 mg po q 12 h × 2 doses, then 200 mg po q 12 h Weight < 40 kg: 200 mg po q 12 h × 2 doses, then 100 mg po q 12 h
	6 mg/kg IV q 12 h × 2 doses followed by 4 mg/kg IV q 12 h
Itraconazole *	200 mg po daily
Posaconazole	300 mg po/IV q 12 h on day 1 followed by 300 mg po/IV daily
Isavuconazole	372 mg po/IV every 8 h × 6 doses followed by 372 mg po/IV daily
Lipid-Formulated Amphotericin B *	3–5 mg/kg IV daily

Abbreviations: mg: milligrams; kg: kilograms; q: every; po: “per os”, by mouth; IV: intravenous. * Requires renal adjustment dose.

## Data Availability

Not applicable.
